# The Effectiveness of Rehabilitation of Occupational Voice Disorders in a Health Resort Hospital Environment

**DOI:** 10.3390/jcm10122581

**Published:** 2021-06-11

**Authors:** Anna Sinkiewicz, Agnieszka Garstecka, Hanna Mackiewicz-Nartowicz, Lidia Nawrocka, Wioletta Wojciechowska, Agata Szkiełkowska

**Affiliations:** 1 Department of Otolaryngology, Audiology and Phoniatrics, University Hospital No. 2, Collegium Medicum, Nicolaus Copernicus University in Toruń, Ujejskiego 75 Street, 85-168 Bydgoszcz, Poland; agnieszka@laryngolog.org (A.G.); hamack@cm.umk.pl (H.M.-N.); lidia.nawrocka@cm.umk.pl (L.N.); 2 Health Resort Hospital in Ciechocinek, Institute of Medical Sciences, Cuiavian University in Włocławek, PlacWolności 1 Street, 87-800 Włocławek, Poland; w.wojciechowska@ksuc.pl; 3 Department of Audiology and Phoniatrics, Institute of Pathology and Physiology of Hearing, Maurycego Mochnackiego 10 Street, 02-042 Warszawa, Poland; a.szkielkowska@ifps.org.pl

**Keywords:** occupational voice disorders, prevention, prophylaxis, teachers, occupational health, voice training, balneotherapy

## Abstract

Background: The aim of this study was to present a rehabilitation program of occupational voice disorders for teachers, conducted in the form of health resort stays, and evaluate its effectiveness depending on job seniority. Methods: The study included 420 teachers who participated in a complex vocal prophylactic and rehabilitation program carried out during a 24-day stay at a health resort hospital. Employment time varied from 4 to 45 years (mean 28.3 years). The participants were divided into three groups: employment time < 21 years (57 teachers), 21–30 years (182 teachers) and > 30 years (181 teachers). All of the subjects underwent maximum phonation time assessment as well as jitter, shimmer and NHR (noise to harmonic ratio) parameters assessment before and after the program; they also underwent perceptual evaluation using the GRBAS scale and voice self-assessment using the VHI-30 scale. Results: The perceptual evaluation using the GRBAS scale and self-report measures of voice function assessed using the VHI scale revealed improvement (*p* < 0.001). The parameters of jitter, shimmer and NHR improved significantly: jitter *p* < 0.001, shimmer *p* < 0.001 and NHR *p* < 0.003. Maximum phonation time increased slightly but significantly (*p* < 0.001). For all of the studied groups regardless of their employment time, maximum phonation time increased (*p* < 0.001). Initially, the lowest values of maximum phonation time were observed in teachers with longer job seniority, which improved after the rehabilitation but remained <15 s. Conclusions: Voice care for teachers is crucial regardless of their job seniority. Early prophylaxis for voice disorders is effective, as the results of rehabilitation are better in teachers with a shorter employment time.

## 1. Introduction

For teachers, the ability to tolerate strain on their vocal organ is essential for safe and comfortable work. Vocal hygiene and stress resistance also play an important role. School teaching is considered to be a profession at a higher risk for developing voice disorders [1,2]. The percentage of teachers with voice problems ranges from 13% [3] to 94% [4]. Lack of sufficient preparation of some teachers for frequent use of their voice at work [5,6,7], difficult working conditions such as noise, working long hours without rest and poor climatic conditions in classrooms result in a higher prevalence of voice disorders than in the general population [8]. The influence of other significant factors on the occurrence of voice disorders, such as age and gender, is also important [9]. Long periods of treatment, surgical interventions and sick leave are associated with high financial costs [2]. This is a widespread social problem involving not only health but also economical aspects [10,11]. It is therefore important to search for effective methods of prevention and rehabilitation programs for occupational voice disorders.

The effectiveness of complex voice rehabilitation programs in ambulatory care has been assessed by many authors [12,13,14,15]. It was observed that vocal hygiene training significantly improves voice quality and reduces disorder symptoms [16,17]. Multicenter efforts to improve quality of care for persons professionally and strenuously using their voice resulted in the development of an interdisciplinary 24-day vocal prophylactic and rehabilitation program conducted in health resort hospitals [18].

The aim of this study was to evaluate the effectiveness of the prevention and rehabilitation program for voice disorders in teachers conducted in a health resort hospital, with analyses of the factors affecting the outcomes.

## 2. Materials and Methods

The study was completed in accordance with the ethical standards of the institutional research committee and principles of the World Medical Association Declaration of Helsinki Ethical Principles for Medical Research involving Human Subjects. Ethical approval for this study was obtained from the Ethics Committee of the Collegium Medicum, Nicolaus Copernicus University. Written informed consent was obtained from patients before the study. 

This program has been implemented in 5 health resort hospitals in Poland, localized in places with a mild climate favorable to the treatment of respiratory diseases. A total of 3685 participants, 3440 female (93.3%) and 245 male (6.7%) participated in a complex rehabilitation program conducted in one health resort hospital between the years 2015–2019. 

The study included teachers who had participated in a 24-day vocal prophylactic and rehabilitation program in 2019. The study group consisted of 420 participants aged 28–64 years (mean 51.4 years) with employment time that varied from 4 years to 45 years (mean 28.3 years). As the teaching profession in Poland is female dominated, all participants included in the study were females who were diagnosed with hyperfunctional dysphonia. Dysphonia had been diagnosed by a referring physician, and the diagnosis was confirmed by an initial phoniatric examination. In order to unify the assessment of voice rehabilitation results and the evaluation of voice acoustic parameters, the study group excluded males and females diagnosed with other diseases, such as glottic insufficiency or chronic hypertrophic laryngitis, which are often permanent voice disorders. Male teachers experience voice disorders less frequently and they constituted only 6.7% of the respondents.

Depending on their employment time, the participants were divided into three groups (Table 1).

**Figure 1:**
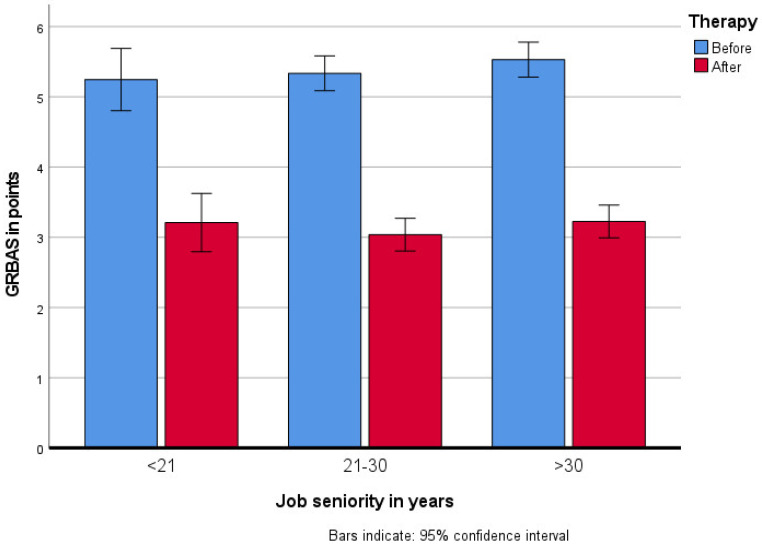
Changes in perceptual voice assessment on the GRBAS scale after the rehabilitation program in groups by their job seniority (the range for the GRBAS scale was 0–15 points).

All of the study participants were subjected to the following initial medical examination: family history taking, laryngological and phoniatric examination. Maximum phonation time (MPT) was obtained as the maximum value of three subsequent trials for each participant to sustain the vowel /a/ for as long as possible using a comfortable pitch and volume [19]:

Perceptual voice evaluation of voice disorders were evaluated using the GRBAS scale: overall grade (G), the degree of hoarse throat intensity; roughness (R), rough voice; breathiness (B), puffing character of voice; asthenia (A), weak voice; and strain (S), voice tension. Each parameter was evaluated on a 4-point scale: 0 (normal), 1 (mild), 2 (moderate), 3 (severe), (Figure 1). The following were also evaluated:

- Videostroboscopy;
- Voice self-assessment: Voice Handicap Index 30 (VHI 30);
- Acoustic analysis of voice;
- Assessment of vocal effort;
- Speech therapists examination;
- Pure tone audiometry.

**Figure 2:**
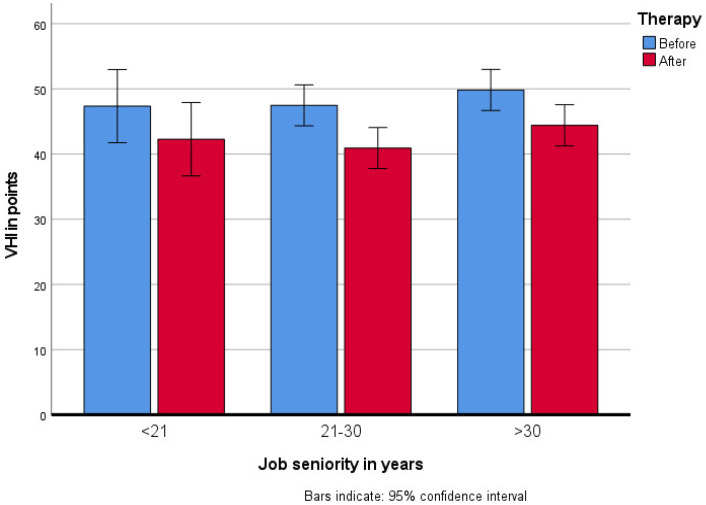
Changes in voice self-assessment on VHI scale after the rehabilitation program in groups by their job seniority.

The assessments were made during the initial examination by a phoniatrist and a speech therapist. The VHI voice self-assessment scale proposed by Jacobson et al. [20] in 1997, the Polish version of which was developed by Pruszewicz et al. in 2004 [21], comprises ten voice disorder variables in three domains: emotional, physical and functional. Patients are requested to note their frequency of each variable on a five-point scale (never, almost never, sometimes, almost ways, always). The score ranges from 30 (unaffected) to 120 (severely affected), (Figure 2) [20,21].

Analysis of voice acoustic parameters (Jitter, Schimmer, NHR) was performed using the DiagnoScope Specialist software [22], before and after the treatment.

The vocal prophylactic and rehabilitation program included educational lectures, voice therapy, physiotherapy and psychotherapy. Educational lectures consisted of vocal hygiene, voice emission mechanisms, voice control, proper voice emission and vocal effort, as well as disorders and laryngeal problems caused by voice abuse, misuse or overuse. The lectures were conducted by a phoniatrist and a speech therapist 5 times per week with durations of 45 min.

Voice rehabilitation consisted of individual and group classes, including relaxation techniques, proper breathing technique, posture, voice emission, articulation and activation of resonators. The aim of the exercises was to eliminate improper breathing, speech and articulation habits, and develop correct habits. Particular attention was paid to voice stabilization and the extension of the phonation time [23]. The exercises were conducted to gain and consolidate the ability to produce a soft voice attack, as well as to enhance the upper vocal tract resonance. A speech therapist conducted individual exercises once a day for 20 min 5 days a week and 30-min group meetings twice a week. 

Physiotherapy included manual therapy, calcium iontophoresis and inhalations. Individual and group psychotherapy was an important part of the program, and focused on stress therapy and stress management techniques. Phoniatric assessment was carried out twice during the program. 

All participants were taken care of by the same team of 2 phoniatrists, 3 speech therapists, 3 physiotherapists and 1 psychologist.

The data were statistically analyzed using the IBM SPSS 25.0.0.1 Toruń, Poland. Analysis of variance was conducted (the therapy effects were tested with the repeated measures; 3 groups depending on their employment time were compared using between group factor in analyses of variance). The Greenhouse–Geisser correction was used when the assumption of sphericity was violated.

## 3. Results

In the perceptual voice evaluation using the GRBAS scale, a statistically significant improvement after therapy (*F*_(1417)_ = 730.33; *p* < 0.001, *η_p_*^2^ = 0.64) was achieved in all voice qualities.

Voice self-assessment on VHI scale improved by more than 6 points after therapy in all the subjects, with a statistical significance of (*F*_(1417)_ = 35.96; *p* < 0.001, *η_p_*^2^ = 0.08).

**Figure 3:**
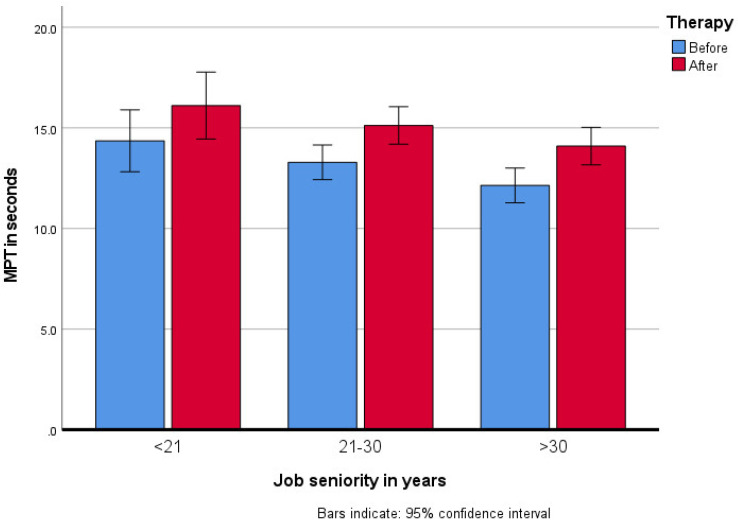
Changes in MPT after the rehabilitation program in groups by their job seniority.

In all groups, regardless of the employment time, MPT prolongation was observed (*F*_(1417)_ = 39.48; *p* < 0.001, *η_p_*^2^ = 0.09). The initial MPT was the shortest in the group with the longest job seniority. After the rehabilitation, MPT improved, as in the other groups, but remained <15 s. Job seniority had the main effect (*F*_(1417)_ = 3.67; *p* = 0.026, *η_p_*^2^ = 0.02). Group comparison showed that MPT in the group with job seniority of up to 20 years differed significantly (*p* = 0.038) from MPT in the patient group with job seniority of over 30 years (Figure 3).

**Figure 4:**
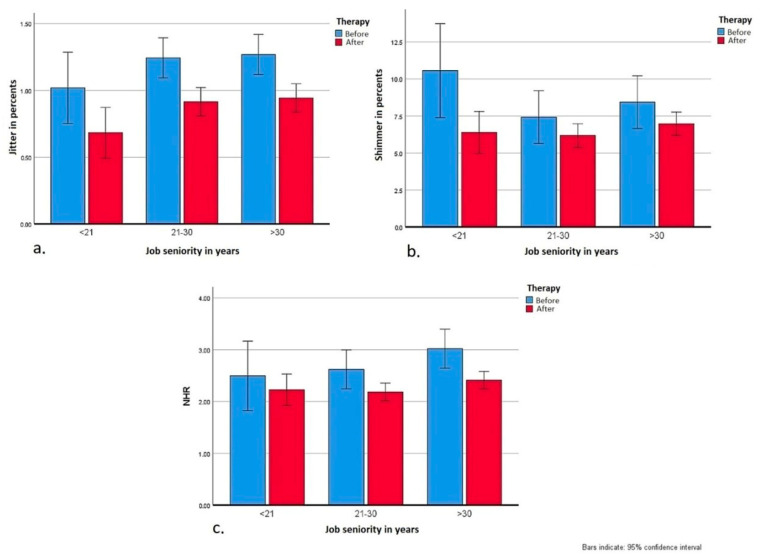
Changes in acoustic parameters after the rehabilitation program in groups by their job seniority: (**a**) jitter; (**b**) shimmer; (**c**) NHR.

In the presented studies, the perceptual voice evaluation using the GRBAS scale, in all features combined, showed a statistically significant improvement and was consistent with both the results of voice self-assessment (VHI questionnaire) and the objective acoustic analyses of the jitter (*F*_(1417)_ = 28.27; *p* < 0.001, *η_p_*^2^ = 0.06), shimmer (*F*_(1417)_ = 10.26; *p* = 0.001, *η_p_*^2^ = 0.02) and NHR parameters (*F*_(1417)_ = 9.12; *p* = 0.003, *η_p_*^2^ = 0.02), (Figure 4). 

## 4. Discussion

Complex voice rehabilitation in the form of stationary health resort treatments sets up conditions for focusing solely on this activity for 24 days, and gives the opportunity to combine systematic exercises, simultaneous physiotherapy and mental relaxation. It is important that the therapy does not cause any voice strain. A break from work without active voice rehabilitation is just a rest, and returning to work means a return to abnormal voice emission patterns and the recurrence of symptoms. Harmful habits, such as an uneconomical breathing pattern practiced for years, lack of control over the laryngeal muscles, speaking too loudly or clearing the throat by grunting, cannot be changed by a one-time recommendation from a physician.

The main problem of rehabilitation psychology is to stimulate the motivation to implement a rehabilitation program [24]. Health resort treatments give the opportunity to start and maintain a healthy lifestyle. This is facilitated by a comfortable sense of well-being related to rest and relaxation, as well as climatic conditions beneficial to the respiratory tract. An important part of the primary and secondary prevention of voice disorders is physical activity, which is often neglected by teachers. A survey by Rosłaniec et al. showed that over 40% of the respondents did not practice physical activity on a regular basis [25]. Other studies have revealed a relationship between the prevalence of voice disorders and a lack of physical activity. Teachers who did not practice physical activity were diagnosed with dysphonia more often than those who exercised three or more times a week [26]. The rehabilitation program offers daily breathing and relaxation exercises. Moreover, participants receive individual recommendations on how to continue exercising at home.

The conditions of health resort-based treatments are particularly conducive to health education, because highly qualified professionals have extensive experience in conducting lectures, talks or interactive workshops. The patients have also free time during their stay, and therefore are positive about participating in educational activities. An educated patient is more independent, has a better quality of life, understands medical recommendations better and turns to specialists for advice less frequently [27].

Data presented on the basis of extensive meta-analyses show that occupational voice disorders are not only caused by the excessive use of voice but are also related to working environments and general health, as well as psychological and sociodemographic factors [9,13,28]. The presented study did not show any worse results from the health resort treatment in patients with comorbidities according to the MPT, jitter, shimmer and NHR acoustic parameters and the GRBAS perceptual evaluation. On the other hand, better initial MPT values were found in teachers with the shortest job seniority, which made their phonation time the longest after the therapy, with a similar improvement in all study groups. The results of the study showed that voice rehabilitation is important in each group, regardless of the employment time; however, the initial breathing capacity and laryngeal muscles are better in younger patients. 

A study by Vaca et al., showed that an age above 50 is associated with an increased risk of voice disorders [29]. Weaker tension of the respiratory and laryngeal muscles can have a negative impact on vocal endurance and voice quality, especially when both deficits occur concomitantly. Voice changes usually refer to difficulties in maintaining the fundamental frequency and shorter phonation time [30]. Patients with the longest work experience are less likely to achieve the desired outcomes of voice rehabilitation, which may not result only from the physiological changes related to age. The study by Rosłaniec et al., showed that teachers over 50 years of age complied with the rules of voice emission and hygiene to a much lesser extent than younger teachers. The VHI voice self-assessment questionnaire is a recognized and useful tool for assessing the progress of voice therapy [31,32,33]. Teachers’ high sensitivity and expectations regarding their own voice make the VHI scale particularly useful in this professional group. However, it is not the numerical value of the VHI test itself that is important but the degree of improvement after treatment [34]. In the study group, after the health resort stay, the voice self-assessment based on the VHI scale improved in all respondents by more than six points (*p* < 0.001).

An improvement in voice parameters after 24 days of an intensive complex rehabilitation program is an expected result. Many authors demonstrated an improvement in the voice of teachers undergoing outpatient rehabilitation [14,35,36].

Therefore, does the presented rehabilitation program allow the intended aim to be achieved more effectively? 

Launching a preventive and rehabilitative program based on a health resort hospital environment requires the initial organization of a diagnostic and rehabilitation base with a team of specialists, and the development of a code of conduct. It is also important to adopt uniform criteria to qualify participants. According to the program assumptions, people with the greatest chance of improving their vocal endurance and voice quality should qualify for the program, which will then enable them to continue their professional career. 

Based on over 5 years of experience with complex health resort-based rehabilitation and the meta-analysis by Byeon, it can be concluded that the essential preconditions for the effectiveness and durability of the treatment are: the condition of the vocal apparatus without permanent disorders, comorbidities that affect the vocal function of the larynx and active participation in all conducted activities [32]. 

Given the benefits of this type of therapy, but also limitations such as a 24-day absence from work and considerable costs of the stay and treatment, it is necessary to develop the optimal, possible frequency of participation in such a rehabilitation program. Repetition of health resort treatments offers a chance to consolidate acquired skills and habits, especially in patients with shorter job seniority.

## 5. Conclusions

In the search for effective methods of prevention and therapy of voice disorders in teachers, it should be recognized that health resort rehabilitation is an attractive form of treatment, as it combines vocal rest with active rehabilitation and health education. An additional advantage of such rehabilitation is climate therapy. Various studies confirmed the purposefulness of voice care at every career stage; however, from the perspective of health and labor economics, early prevention is more appropriate because there is a better chance for voice regeneration for people with shorter work experience. 

The co-financing of such rehabilitation is also of great importance, as its multidisciplinarity is associated with considerable costs. In the end, however, the benefits outweigh the otherwise possible expenses related to illness treatment, sick leave and other problems related to the continuation of participants’ professional careers.

## Figures and Tables

**Table 1:** Employment time.

Employment Time (Years)	Number of Patients	Mean Employment Time (Years)
<21	57	15.3
21–30 >30	182 181	26.8 34.0
Total	420	28.3

## Data Availability

All data used to support the finding of this study are available from the corresponding author upon request.
